# The complete chloroplast genome and phylogenetic analysis of *Corydalis fangshanensis* W.T. Wang ex S.Y. He (Papaveraceae)

**DOI:** 10.1080/23802359.2021.1987172

**Published:** 2021-10-08

**Authors:** Zhiying Yu, Tianhua Zhou, Niya Li, Dong Wang

**Affiliations:** a School of Life Sciences, Central China Normal University, Wuhan, Hubei Province, P. R. China; b Bio-Resources Key Laboratory of Shaanxi Province, School of Life Sciences, Shaanxi University of Technology, Hanzhong, Shaanxi Province, P. R. China; c Wuhan Sinoeco Ecological Science & Technology Co., Ltd., Wuhan, Hubei Province, P. R. China

**Keywords:** *Corydalis fangshanensis*, chloroplast genome, phylogenetic analysis

## Abstract

The complete chloroplast (cp) genome of *Corydalis fangshanensis* W.T. Wang ex S.Y. He, a Chinese endemic plant with limestone-specific distribution was first reported. The cp genome was circular in structure and 192,554 bp in length, consisting of a large single copy region (LSC, 98,393 bp), two inverted repeat regions (IRs, 42,263 bp), and a small single copy region (SSC, 9,635 bp). The overall GC content of the genome was 40.26%. It encoded 112 unique genes, including 78 protein-coding genes, 30 tRNA genes, and 4 rRNA genes. Phylogenetic analysis resolved *C*. *fangshanensis* was closely related to *C. saxicola* G.S. Bunting within *Corydalis* sect. *Thalictrifoliae* (Fedde) Lidén, in line with morphological character-based taxonomy. Our result provides informative data for studying the taxonomy, phylogeny and ecology of *Corydalis*, especially species with specific-limestone distribution and also for studying the adaptive evolution in plants.

*Corydalis* DC. is the most species-rich genus in Papaveraceae and comprises ca. 465 species. Most of the species distributed in China with 357 species of which 262 are endemic (Zhang et al. 2008). Some species of the genus occur within specialized habitats of limestone cliffs, such as members belonging in *Corydalis* sect. *Talictrifoliae* (Fedde) Lidén (i.e., *C. fangshanensis* W.T. Wang ex S.Y. He, *C. latiloba* (Franch.) Handel-Mazz., *C. saxicola* G.S. Bunting, *C. tomentella* Franch., and *C. wilsonii* N.E. Br. (Wu et al. 1999; Zhang et al. 2008). Chloroplasts genomes regarding gene content and order are highly conserved, with the potential for the understanding of the taxonomy, phylogeny and evolution of plants (Ahlert et al. 2003; Moore et al. 2010). But less than ten species of *Corydalis* genomes are publicly available in NCBI to data (Wu et al. 2020; Xu and Wang 2020, 2021; Liu et al. 2021; Ren et al. 2021). *Corydalis fangshanensis* W.T. Wang ex S.Y. He was described on the basis of collection (T.N. Liou 8318, holotype) from Shangfan mountain, Fangshan district, Beijing (He et al. 1984). It is a species endemic to China and the plants readily grow in limestone habitats. It is famous for one of the ‘three wonderful wild flowers in Beijing’ (another two species are *Clematis acerifolia* Maxim. and *Oresitrophe rupifraga* Bunge). Yet, the complete chloroplast genome of *C. fangshanensis* is lacking.

In this study, the material used for *C. fangshanensis* was retrieved from its wild population (Luliang mountains, Shanxi, China, 37°12′N, 111°13′E, 1317 m). Voucher specimens and DNA (D. Wang 190019) were deposited in the herbarium of Central China Normal University (CCNU, acronyms of herbaria according to Thiers 2021). In the wild, the fresh leaves were dried using silica gel and preserved at −20 °C until DNA extraction. Total genomic DNA was isolated using the modified CTAB method (Doyle and Doyle 1987), and followed by inserting an average size of 350 bp fragments, then constructed, and sequenced using Illumina NovaSeq 6000 at Novogene (Tianjing, China). In total, 12,390,017 raw reads were generated, and then filtered using fastp v0.20.1 (Chen et al. 2018) to get high-quality clean data. A total of 12,244,806 cleaned reads were assembled using GetOrganelle v1.6.2 (Jin et al. 2020) and the complete cp genome was annotated using PGA (Qu et al. 2019), both with the plastome of *C. inopinata* (NC_052866) as reference.

To elucidate the systematic position of *C*. *fangshanensis,* we used 97 shared genes among *C*. *fangshanensis* and another six publical plastomes of *Corydalis* species, together with *Lamprocapnos spectabilis* (L.) Fukuhara (NC_039756) and *Papaver somniferum* L. (NC_029434) as outgroups. These genes were aligned with default parameters using MAFFT v.7 (Katoh and Standley 2013). The best-fit nucleotide substitution model for Bayesian inference (BI) analysis was determined by jModeltest v2.1.10 (Darriba et al. 2012) using the Akaike information criterion (AIC). Maximum likelihood (ML) analysis was performed using RAxML v8.2.12 (Stamatakis 2014) with 1000 bootstrap replicates and the GTRGAMMA model. BI analysis was conducted using MrBayes v3.2.7 (Ronquist et al. 2012, GTR + I + G model, ngen = 1,000,000, samplefreq = 100, nchains = 4, burnin = 2500).

The complete cp genome of *C*. *fangshanensis* was 192,554 bp in length with a typical angiosperm quadripartite structure, containing a pair of inverted repeat (IR) regions of 42,263 bp each, separated by a large single-copy (LSC) region of 98,393 bp and a small single-copy (SSC) region of 9,635 bp. The GC content of the whole chloroplast genome, LSC, SSC, and IR regions were 40.26%, 39.17%, 35.22% and 42.10%, respectively. A total of 112 unique genes were predicted, including 78 protein-coding genes, 30 tRNA genes, and four rRNA genes. Most of these genes were single-copy genes, however, twenty-five genes were duplicated in the IR region, including 13 protein-coding genes (*rpl23*, *ycf2*, *rps12*, *rps7*, *ndhB*, *ndhF*, *rpl32*, *ccsA*, *ndhD*, *psaC*, *ndhE*, *ndhG*, and *ndhI*), eight tRNAs (*trnI-CAU*, *trnL-CAA*, *trnR-ACG*, *trnA-UGC*, *trnI-GAU*, *trnV-GAC*, *trnN-GUU*, and *trnL-UAG*), and four rRNAs *(rrn4.5*, *rrn5*, *rrn23*, and *rrn16*). In 112 unique genes, 15 genes (six tRNA genes and nine protein-coding genes) contained one intron, whereas two genes (*ycf3* and *rps12*) contained double introns, even one gene (*clpP*) contained three introns. Furthermore, the *accD* gene was absent. With regard to gene content, GC content and gene order of *C. fangshanensis*, we found that they were similar to those of *C. saxicola*.

Phylogenetic analysis generated a well-supported phylogenetic tree, with all nodes having Maximum likelihood (ML) bootstrap support values =100 and Bayesian inference (BI) posterior probabilities =1 (Figure 1). The ML and BI analysis both indicated that *C*. *fangshanensis* was closely related to *C. saxicola* within *Corydalis* sect. *Thalictrifoliae* (Fedde) Lidén, in agreement with morphological character-based taxonomy. The genomic data provided here can aid the ecology and evolution research in *Corydalis*, especially species with specific-limestone distribution.

**Figure 1.**
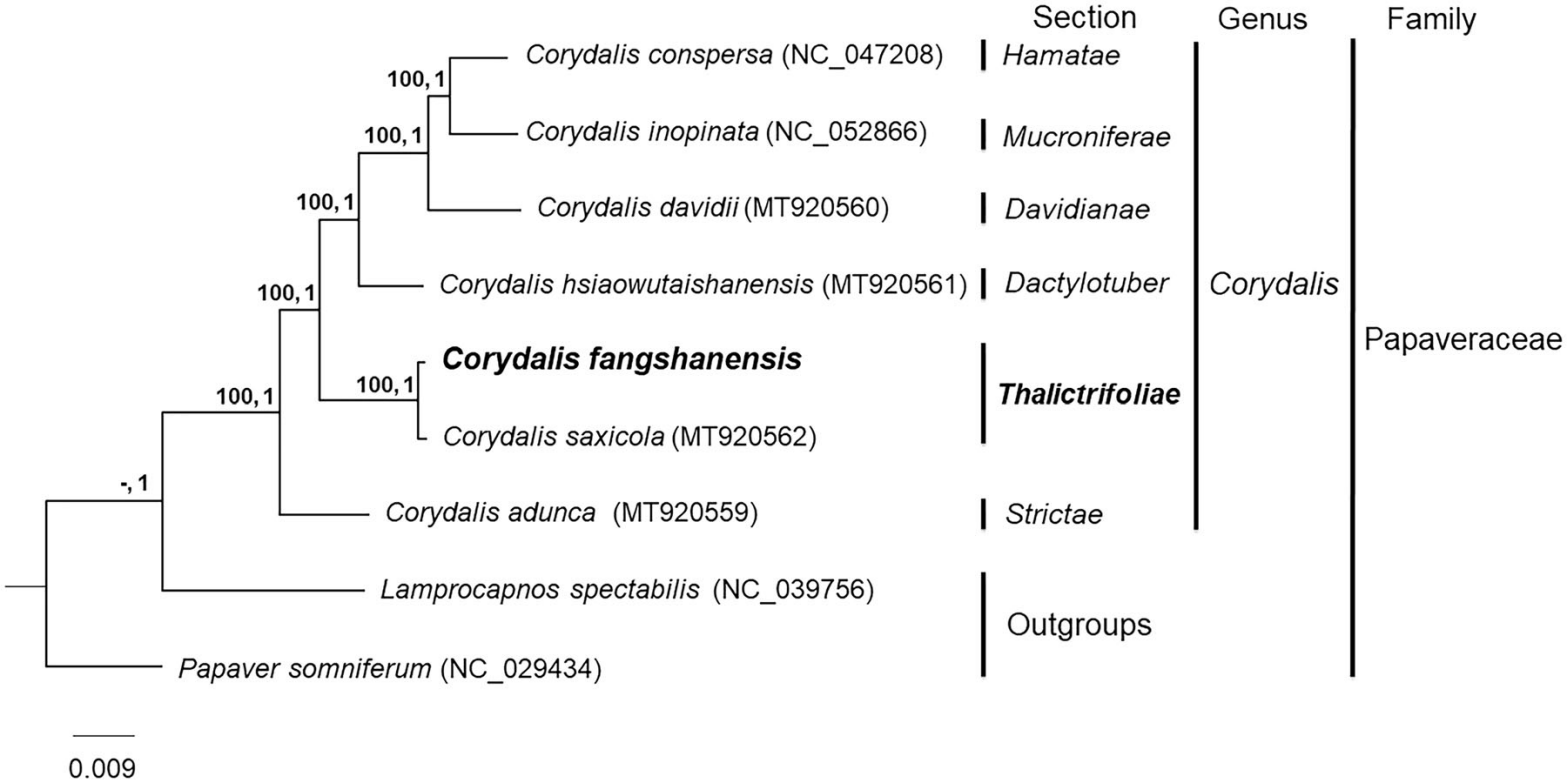
Phylogenetic relationship of *C. fangshanensis* with another six species of *Corydalis*, with ML bootstrap values (left) and BI posterior probability (right) indicated near the nodes.

## Data Availability

The data that support the findings of this study are openly available in National Center for Biotechnology Information (NCBI) at https://www.ncbi.nlm.nih.gov, reference number MZ440305. The associated BioProject, SRA, and Bio-Sample numbers are PRJNA739560, SRR14871404, and SAMN19795489, respectively.
